# Multidisciplinary team meetings improve survival in patients with esophageal cancer

**DOI:** 10.1093/dote/doae061

**Published:** 2024-08-09

**Authors:** Mats Lindblad, Christine Jestin, Jan Johansson, David Edholm, Gustav Linder

**Affiliations:** Department of Clinical Science, Intervention and Technology, Karolinska Institute, Stockholm, Sweden; Department of Surgical Sciences, Uppsala University, Uppsala, Sweden; Department of Surgery, Lund University, Lund, Sweden; Department of Surgery, Linköping University, Linköping, Sweden; Department of Surgical Sciences, Uppsala University, Uppsala, Sweden

**Keywords:** cancer, esophageal, survival

## Abstract

Multidisciplinary team meetings (MDTs) are recommended for patients with esophageal cancer. Improved staging, timeliness to surgery and better adherence to guidelines have been attributed to MDTs, but there are few studies published on the MDTs’ effect on survival. All patients with esophageal cancer in Sweden between 2006 and 2018 were grouped according to whether they had been discussed at an MDT as part of their clinical pathway. Factors affecting group allocation were explored with multivariable logistic regression, and the impact of MDT on survival was studied with Cox-regression and the Kaplan–Meier estimator. Of 6837 included patients, 1338 patients (20%) were not discussed at an MDT. Advanced age (80–90 years; odds ratio [OR] 0.25, 0.16–0.42 (95% confidence interval)) and clinical stage IVb (OR 0.65, 0.43–0.98) decreased the probability of being presented at an MDT, whereas high education level (OR 1.31, 1.02–1.67), being married (OR 1.20, 1.01–1.43), squamous histology (OR 1.50, 1.22–1.84) and later year of diagnosis (OR 1.33, 1.29–1.37 per year) increased the probability of an MDT. In multivariable adjusted analysis, MDT discussion was associated with improved survival (hazard ratios 0.72, 0.66–0.78) and median survival increased from 4.5 to 10.7 months. MDTs were associated with improved survival for esophageal cancer patients. Elderly patients with advanced disease and poor socioeconomic status were less likely to be presented at an MDT, but had clear survival-benefits if they were discussed in a multidisciplinary setting.

## INTRODUCTION

Modern curatively intended treatment of esophageal cancer is often multimodal including a combination of oncologic and surgical treatment. Present treatment regimens for esophageal cancer include perioperative chemotherapy or neoadjuvant chemoradiotherapy, in combination with surgical resection, with or without adjuvant immunotherapy, which has been shown to improve outcomes in comparison to surgical treatment alone.^1^^,^^2^

In addition to multimodal treatment including surgical resection, definitive chemoradiotherapy is an option for cure in selected patients with squamous cell carcinoma. In patients with non-curable disease, or for patients not fit for curative treatment, palliative oncologic treatment might be considered.

The multimodal treatment regimens are complex and require experienced evaluation for optimal patient selection and detailed planning and coordination among health care professionals. In multidisciplinary team meetings (MDTs) health care professionals from different disciplines discuss patients to decide on the best treatment recommendation for each patient. In esophageal cancer, such MDTs often include surgeons, radiologists, oncologists, pathologists and dedicated cancer nurse specialists but other disciplines and professions may also be represented, for example, gastroenterologists, rehab physicians, dieticians and more.

Swedish national guidelines state that patients diagnosed with esophageal cancer should be discussed in a multidisciplinary setting and that at the MDT there must be at least one surgeon, one oncologist, one radiologist and one contact nurse present for the requirements to be met.^3^ In Sweden, the MDT services are centralized according to the six geographical healthcare regions of the country, which each harbors a university hospital for surgical management of gastroesophageal cancer. Each university hospital hosts a regional MDT taking referrals from county hospitals who in most circumstances attend the MDT online. The uptake population ranges from 1 to 2.5 million for each center. A national MDT for highly selected patients and second opinions has also recently been made available.

There remains a paucity whether multidisciplinary management in esophageal cancer translates to a survival benefit. There have been no randomized trials investigating the role of the MDT on survival although some studies have shown treatment related benefits attributed to the MDT such as increased adherence to guidelines and improved timeliness to surgical treatment.^4^ Improved survival associated with the MDT has previously been reported for esophageal cancer, albeit from either small retrospective single center studies^5^^,^^6^ or one larger Asian study limited to patients receiving curative treatment.^7^

The aim of this study was to assess patient-related factors associated with being presented at an MDT as well as evaluate the role of the MDT on survival for esophageal cancer patients in a western setting.

##  METHODS

### Study design

A register-based retrospective study was conducted including all patients in the Swedish Registry for Esophageal- and Gastric Cancer (NREV) diagnosed with esophageal- or gastroesophageal junction cancer (International Classification of Disease 10th version [ICD-10]: C15.3-C15.9, C16.0A, C16.0B, C16.0X) in Sweden between 1 January 2006 and 26 February 2018. NREV is a comprehensive registry with high validity that includes more than 95% of patients diagnosed with esophageal cancer in Sweden.^8^ Additional data from well-described and validated registries, including the Swedish Prescribed Drug Register,^9^ Cause-Of-Death Register,^10^ Cancer Register,^11^ Patient Register,^12^ and the Longitudinal Integrated Database for Health Insurance and Labor Market Studies^13^, were utilized to create a study specific database for analysis. The cross-linking of registries was made possible through a unique 10-digit personal identification number given to all Swedes at birth. All data from above registries were extracted in 2020.

### MDT—exposure

Data on the treatment decision pathway were retrieved from the NREV. Since the start of NREV, in 2006, the register has included a mandatory variable with information on whether the treatment recommendation was made at an MDT or not. The variable is reported on the diagnostic form, which concludes the diagnostic workup after a decision on treatment recommendation has been made. The exposure in this study, the variable MDT (yes/no), has an accuracy of 93.8%.^8^ The definition of an MDT, according to the NREV, was in keeping with national guidelines as presented above.^3^ In Sweden, patients are not present during MDT conferences for esophageal cancer.

### Survival—outcome

Survival was calculated from the date of diagnosis until death or censored at the time of emigration or end of follow-up (17 April 2018), whichever came first. Complete follow-up was achieved due to the nationwide and complete coverage of both the Total Population register as well as the Cause of Death Register.

### Confounding factors and covariates

Information on patients- and tumor characteristics, treatment related information, educational data as well as marital status were retrieved from the databases described above. For each patient a Charlson Comorbidity Index (CCI) was calculated.^14^

A directed acyclic graph model was utilized to identify associating- and biasing pathways to discern confounding factors that should be included in the multivariable analyses. The following covariates were identified to possibly be associated with the presence of an MDT and thus included in the multivariable models: age (below 50 and thereafter in 10-year intervals up to 90 and above), sex, comorbidities (CCI; 0, 1–2, ≥3), clinical stage according to the 8th edition of the AJCC/UICC Tumor Nodal Metastasis (TNM) classification system (stages I–IVb), education level (<9 years, 10–12 years or > 12 years of schooling), marital status (married/unmarried), year of diagnosis (per year or in tertiles of the study period), histopathology (adenocarcinoma or squamous cell carcinoma) and treatment intent (curative treatment or palliative treatment/best supportive care).

### Statistical analysis

Baseline data and patient characteristics were presented and analyzed with descriptive statistics. Covariates and factors associated with the patient being presented at an MDT were explored with uni- and multi-variable logistic regression presented as odds ratios (OR) with 95% confidence intervals (CI).

Survival probability was displayed according to the Kaplan–Meier method and Cox proportional hazard models were used to assess the effect of an MDT when adjusting for multiple variables and was presented as hazard ratios (HR) with 95% CI. Sub-group analysis was performed with separate Cox models and Kaplan–Meier curves to assess the impact of the MDT in certain risk-groups where multidisciplinary management was hypothesized to be less frequent and possibly of considerable importance. These risk groups included patients with advanced age (>80 years old), advanced clinical disease stage (stage IV) or advanced comorbidities (CCI 3 or above). To explore immortal time bias additional survival analysis was performed by changing the index date to 21 days after diagnosis (median time from diagnosis to MDT).

All statistical analysis was performed with STATA© version 15.1 (StataCorp, College Station, TX, USA).

Ethical approval was obtained from the regional ethical review board in Stockholm (2013/596-31/3 and 2016/1486-32).

## RESULTS

### Patients

All 6837 patients identified in NREV as diagnosed with esophageal- or gastroesophageal junction cancer during the study period were included in the study. Patient- and tumor characteristics are summarized in Table 1. A majority of patients were male and the median age at diagnosis was 70 years. More than 2/3 had advanced esophageal cancer with tumor stage III or above and slightly less than 60% were planned for palliative treatment.

**Table 1:** Baseline characteristics of 6837 patients with esophageal cancer between 2006 and 2018 in Sweden, grouped according to the presence of an MDT

		No MDT *n* = 1338	MDT *n* = 5276	Missing *n* = 223	Total *n* = 6837
Sex, no (%)					
	Female	384 (28.7%)	1322 (25.1%)	54 (24.2%)	1760 (25.7%)
	Male	954 (71.3%)	3954 (74.9%)	169 (75.8%)	5077 (74.3%)
Age, median (IQR)					
	Years	76 (66, 84)	69 (62, 76)	69 (61, 77)	70 (63, 78)
CCI No (%)					
	0	488 (36.5%)	2150 (40.8%)	103 (46.2%)	2741 (40.1%)
	1–2	192 (14.3%)	719 (13.6%)	40 (17.9%)	951 (13.9%)
	3 or above	658 (49.2%)	2407 (45.6%)	80 (35.9%)	3145 (46.0%)
Clinical stage, no (%)					
	Stage 0	19 (1.4%)	33 (0.6%)	1 (0.4%)	53 (0.8%)
	Stage I	38 (2.8%)	152 (2.9%)	7 (3.1%)	197 (2.9%)
	Stage II	115 (8.6%)	758 (14.4%)	22 (9.9%)	895 (13.1%)
	Stage III	150 (11.2%)	1576 (29.9%)	43 (19.3%)	1769 (25.9%)
	Stage IVa	38 (2.8%)	509 (9.6%)	11 (4.9%)	558 (8.1%)
	Stage IVb	516 (38.6%)	1631 (30.9%)	46 (20.6%)	2193 (32.1%)
	Missing	462 (34.5%)	617 (11.7%)	93 (41.7%)	1172 (17.1)
Clinical T-stage, no (%)					
	Tis/HGD	29 (2.2%)	42 (0.8%)	3 (1.3%)	74 (1.1%)
	T1	64 (4.8%)	192 (3.6%)	9 (4.0%)	265 (3.9%)
	T2	159 (11.9%)	820 (15.5%)	29 (13.0%)	1008 (14.7%)
	T3	367 (27.4%)	2637 (50.0%)	62 (27.8%)	3066 (44.9%)
	T4a	165 (12.3%)	724 (13.7%)	27 (12.1%)	916 (13.4%)
	T4b	20 (1.5%)	124 (2.4%)	1 (0.4%)	145 (2.1%)
	Missing	534 (39.9%)	737 (14.0%)	92 (41.3%)	1363 (19.9%)
Clinical N-stage, no (%)					
	N0	404 (30.2%)	1991 (37.7%)	42 (18.8%)	2437 (35.6%)
	N1	330 (24.7%)	1891 (35.8%)	83 (37.2%)	2304 (33.7%)
	N2	91 (6.8%)	613 (11.6%)	8 (3.6%)	712 (10.4%)
	N3	49 (3.7%)	419 (7.9%)	7 (3.1%)	475 (6.9%)
	Missing	464 (34.7%)	362 (6.9%)	83 (37.2%)	909 (13.3%)
Clinical M-stage, no (%)					
	M0	605 (45.2%)	3503 (66.4%)	106 (47.5%)	4214 (61.6%)
	M1	516 (38.6%)	1631 (30.9%)	46 (20.6%)	2193 (32.1%)
	Missing	217 (16.2%)	142 (2.7%)	71 (31.8%)	430 (6.3%)
Histopathology, no (%)					
	Adenocarcinoma	1006 (75.2%)	3624 (68.7%)	145 (65.0%)	4775 (69.8%)
	Squamous cell carcinoma	332 (24.8%)	1652 (31.3%)	78 (35.0%)	2062 (30.2%)
Year of diagnosis, no (%)					
	2005–2009	715 (53.4%)	1250 (23.7%)	184 (82.5%)	2149 (31.4%)
	2010–2013	435 (32.5%)	1809 (34.3%)	16 (7.2%)	2260 (33.1%)
	2014–2018	186 (13.9%)	2202 (41.7%)	21 (9.4%)	2409 (35.2%)
	Missing	2 (0.1%)	15 (0.3%)	2 (0.9%)	19 (0.3%)
Marital status, no (%)					
	Non-married	760 (56.8%)	3146 (59.6%)	115 (51.6%)	4021 (58.8%)
	Married	578 (43.2%)	2130 (40.4%)	108 (48.4%)	2816 (41.2%)
Education level, no (%)					
	Low (<9 years)	527 (39.4%)	1964 (37.2%)	75 (33.6%)	2566 (37.5%)
	Intermediate (10–12 years)	442 (33.0%)	2138 (40.5%)	72 (32.3%)	2652 (38.8%)
	High (>12 years)	157 (11.7%)	965 (18.3%)	25 (11.2%)	1147 (16.8%)
	Missing	212 (15.8%)	209 (4.0%)	51 (22.9%)	472 (6.9%)
Planned treatment, no (%)					
	Upfront surgery	171 (12.8%)	681 (12.9%)	153 (68.6%)	1005 (14.7%)
	Neoadjuvant therapy + surgery	36 (2.7%)	1293 (24.5%)	3 (1.3%)	1332 (19.5%)
	Definitive chemoradiotherapy	24 (1.8%)	490 (9.3%)	5 (2.2%)	519 (7.6%)
	Palliation	1107 (82.7%)	2812 (53.3%)	62 (27.8%)	3981 (58.2%)

Clinical T-, N- and M-stage calculated according to the 8th edition of the AJCC/UICC TNM classification system

**Fig. 1:**
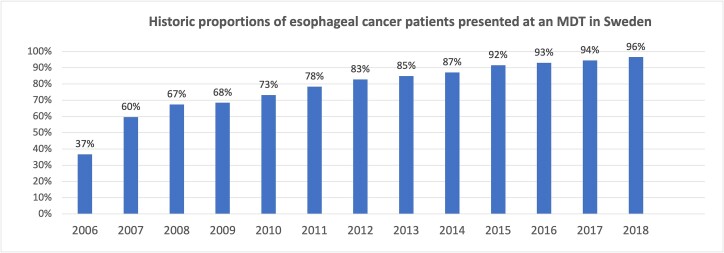
Historic proportions of patients diagnosed with esophageal cancer in Sweden that were presented at an MDT.

Of the 6837 patients, 5276 (77.2%) were presented at an MDT for recommendation on treatment whereas 1338 (19.6%) were not discussed in such a multidisciplinary setting. Information regarding MDT was missing in 223 patients (3.2%), Table 1. Presenting patients at an MDT became increasingly common during the study period (Fig. 1).

Patients that had treatment recommendations from an MDT were more often allocated to treatment with curative intent compared to patients that were not discussed in a multidisciplinary setting (46.7% vs. 17.3%). The proportion of patients allocated to upfront surgery was similar in the two groups while trimodal treatment and definitive chemoradiotherapy were more common in the group of patients that were presented at an MDT, Table 1. Active palliative treatment plans, made at the time of diagnosis, with chemo- and/or radiotherapy or palliative interventions, were more common in patients presented at an MDT. In patients where curative treatment was not possible, active palliation was planned in 69.5% of patients with an MDT and only in 30.0% of patients without an MDT, Table 1.

### Factors associated with an MDT

Patients discussed at an MDT were generally younger, had fewer comorbidities, less advanced disease and presented more often with squamous cell carcinoma, Tables 1 and 2.

**Table 2:** OR with 95% confidence limits (CI) from logistics regression model for the outcome case presented to an MDT

	Multivariate		
	OR	(95% CI)	*p*
Sex			
Female	1.00	(Ref.)	—
Male	1.08	(0.89–1.32)	0.437
Age at diagnosis (years)			
<50	1.00	(Ref.)	—
50–59	1.01	(0.61–1.66)	0.981
60–69	0.87	(0.55–1.40)	0.574
70–79	0.63	(0.39–1.02)	0.059
80–89	0.25	(0.16–0.42)	<0.001
90 and above	0.08	(0.04–0.16)	<0.001
Marital status			
Unmarried	1.00	(Ref.)	—
Married	1.20	(1.01–1.43)	0.043
Education level			
Low (<9 years)	1.00	(Ref.)	—
Intermediate (10 – 12 years)	1.04	(0.86–1.25)	0.673
High (>12 years)	1.31	(1.02–1.67)	0.036
CCI score			
0–1	1.00	(Ref.)	—
2	0.92	(0.71–1.19)	0.523
3 or above	0.84	(0.70–1.02)	0.074
Histopathology			
Adenocarcinoma	1.00	(Ref.)	—
Squamous	1.50	(1.22–1.84)	<0.001
Clinical disease stage			
Stage 0	0.37	(0.17–0.81)	0.012
Stage I	1.00	(Ref.)	—
Stage II	1.45	(0.93–2.29	0.104
Stage III	2.66	(1.72–4.11)	<0.001
Stage IVa	2.08	(1.21–3.58)	0.008
Stage IVb	0.65	(0.43–0.98)	0.043
Year of diagnosis (continuous)			
per each additional year from 2006	1.33	(1.29–1.37)	<0.001

Clinical T-, N- and M-stage calculated according to the 8th edition of the AJCC/UICC TNM classification system

Analysis with multivariable logistic regression, utilizing the presence of an MDT as outcome, showed associations between the presence of an MDT and several factors (Table 2): Older patients had a reduced chance of being presented at MDT (age 80–90; OR 0.25, 0.16–0.42 95% CI and age above 90; OR 0.08, 0.04–0.16 95% CI).

Socioeconomic factors associated with an increased chance of being presented at an MDT were high education level (OR 1.31, 1.02–1.67 95% CI) and being married (OR 1.20, 1.01–1.43 95% CI). Tumor factors increasing the likelihood of being presented at an MDT were squamous cell carcinoma histology (OR 1.50, 1.22–1.84 95%CI) and advanced, but non-metastatic, clinical tumor stage (stages III and IVa; OR 2.66 and 2.08, respectively, 1.72–4.11 and 1.21–3.58 95%CI) while stage IVb was associated with reduced chance of being presented at an MDT (OR 0.65, 0.43–0.98 95%CI). Additionally, with each progressing year of the study the probability of an MDT increased with roughly one third (OR 1.33 per year, 1.29–1.37 95%CI).

### MDT and survival

**Fig. 2:**
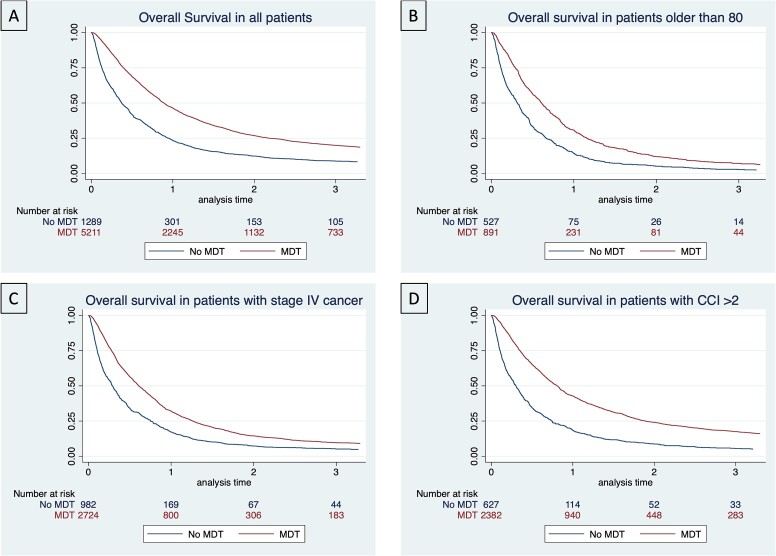
Kaplan Meier curves displaying survival in esophageal cancer patients, conditional on the presentation to an MDT. Survival is presented for (A) the entire cohort, (B) patients older than 80 years, (C) patients with stage IV cancer and (D) patients with a Charleston comorbidity score of 3 or above.

The unadjusted 3-year overall survival was 20.0% in patients presented at an MDT and 8.7% in patients without an MDT. Corresponding median survival was 10.7 and 4.5 months (Fig. 2A). The sub-group analysis of patients older than 80 displayed a 3-year survival of 7.1% for patients with an MDT and 2.8% in patients without MDT assessment which translated to a median survival of 7.3 and 3.8 months, respectively (Fig. 2B). In patients with advanced disease stage or with advanced comorbidities findings were similar and in favor of MDT presentation. The 3-year survival for stage IV patients was 9.6% and 5.1% (MDT/no MDT) and for patients with CCI > 2 17.5% versus 5.5% (MDT/no MDT), respectively (Fig. 2C and D).

In the 6837 patients diagnosed with esophageal cancer, multivariable Cox regression with the outcome death from any cause demonstrated the MDT to be a significant predictor of survival (HR 0.72, 0.66–0.78 95%CI). Additional factors associated with improved survival were being married (HR 0.93, 0.87–0.99 95%CI) as well as higher education level (intermediate; HR 0.92, 0.86–0.99 95%CI and high; HR 0.86, 0.78–0.94 95%CI). Poorer prognosis in the cohort was identified for men (HR 1.11, 1.03–1.19 95%CI), older patients, more comorbid patients, patients with advanced clinical tumor stage, squamous cell carcinoma histology (HR 1.41, 1.25–1.69 95%CI) and for patients allocated to palliative treatment or best supportive care (2.47, 2.25–2.70 95%CI), Table 3.

**Table 3:** HR with 95% CI from Cox Proportional hazards models in 6837 patients diagnosed with esophageal cancer for the outcome death by any cause

		All patients		
		Multivariate		
		HR	(95% CI)	*p*
MDT				
	No	1.00	(Ref.)	—
	Yes	0.72	(0.66–0.78)	<0.001
Sex				
	Female	1.00	(Ref.)	—
	Male	1.11	(1.03–1.19)	0.006
Age at diagnosis (years)				
	<50	1.00	(Ref.)	—
	50–59	1.21	(1.01–1.46)	0.046
	60–69	1.30	(1.09–1.55)	0.003
	70–79	1.54	(1.29–1.84)	<0.001
	80–89	1.69	(1.40–2.05)	<0.001
	90 and above	3.11	(2.34–4.14)	<0.001
Marital status				
	Unmarried	1.00	(Ref.)	—
	Married	0.93	(0.87–0.99)	0.025
Education level				
	Low (<9 years)	1.00	(Ref.)	—
	Intermediate (10 – 12 years)	0.92	(0.86–0.99)	0.025
	High (>12 years)	0.86	(0.78–0.94)	0.001
CCI score				
	0–1	1.00	(Ref.)	—
	2	0.94	(0.86–1.04)	0.242
	3 or above	1.13	(1.06–1.21)	<0.001
Histopathology				
	Adenocarcinoma	1.00	(Ref.)	—
	Squamous	1.24	(1.15–1.33)	<0.001
Clinical disease stage				
	Stage 0	1.00	(Ref.)	—
	Stage I	0.99	(0.64–1.54)	0.968
	Stage II	1.55	(1.04–2.32)	0.033
	Stage III	2.17	(1.46–3.23)	0.004
	Stage IVa	2.47	(1.65–3.72)	<0.001
	Stage IVb	3.34	(2.24–4.99)	<0.001
Year of diagnosis				
	2005–2009	1.00	(Ref.)	—
	2010–2013	1.09	(1.01–1.18)	0.033
	2014–2018	0.99	(0.91–1.08)	0.887
Treatment allocation				
	Curative treatment	1.00	(Ref.)	—
	Palliation	2.47	(2.25–2.70)	<0.001

Clinical T-, N- and M-stage calculated according to the 8th edition of the AJCC/UICC TNM classification system

Association with improved outcome

Association with poorer outcome

In the Cox regression models for sub-group analysis of risk group patients, the presence of an MDT was the only factor significantly associated with improved survival (HR 0.59 for patients >80 years old, HR 0.71 for patients with stage IV disease and HR 0.62 for patients with CCI 3 or above). Predictors of poorer outcome were in keeping with the main Cox regression analysis with the addition that poorer outcome was also identified for patients diagnosed in the latter two tertiles of the study period, Table 4. A further selected sub-group analysis of patients with all the above risk factors (age > 80, stage IV disease and CCI 3 or above) confirmed that, even in this group of particularly smitten patients, the MDT was still associated with improved survival, HR 0.59, Supplementary Table 1.

**Table 4:** HR with 95% CI from Cox Proportional hazards models for the outcome death by any cause. Stratified on advanced age, stage IV disease and advanced comorbidities, CCI 3 or above

	Patients with age ≧ 80			Patients with stage IV disease			Patients with CCI ≧ 3		
	Multivariate			Multivariate			Multivariate		
	HR	(95% CI)	*p*	HR	(95% CI)	*p*	HR	(95% CI)	*p*
MDT									
No	1.00	(Ref.)	—	1.00	(Ref.)	—	1.00	(Ref.)	—
Yes	0.59	(0.49–0.70)	<0.001	0.71	(0.65–0.78)	<0.001	0.62	(0.54–0.71)	<0.001
Sex									
Female	1.00	(Ref.)	—	1.00	(Ref.)	—	1.00	(Ref.)	—
Male	1.03	(0.87–1.21)	0.734	1.12	(1.03–1.22)	0.011	1.16	(1.04–1.29)	0.008
Age at diagnosis (years)									
<50				1.00	(Ref.)	—	1.00	(Ref.)	—
50–59				1.28	(1.02–1.59)	0.030	0.84	(0.59–1.18)	0.306
60–69				1.25	(1.01–1.53)	0.036	1.01	(0.73–1.39)	0.962
70–79				1.47	(1.19–1.81)	<0.001	1.24	(0.90–1.70)	0.191
80–89				1.42	(1.14–1.77)	0.002	1.33	(0.96–1.85)	0.089
90 and above				1.93	(1.45–2.58)	<0.001	2.19	(1.44–3.33)	<0.001
Marital status									
Unmarried	1.00	(Ref.)	—	1.00	(Ref.)	—	1.00	(Ref.)	—
Married	1.00	(0.84–1.17)	0.966	0.94	(0.87–1.01)	0.104	1.02	(0.93–1.12)	0.651
Education level									
Low (<9 years)	1.00	(Ref.)	—	1.00	(Ref.)	—	1.00	(Ref.)	—
Intermediate (10 – 12 years)	0.97	(0.82–1.14)	0.699	0.94	(0.87–1.02)	0.150	0.94	(0.85–1.04)	0.241
High (>12 years)	0.96	(0.77–1.19)	0.695	0.91	(0.82–1.01)	0.076	0.91	(0.80–1.04)	0.168
CCI score									
0–1	1.00	(Ref.)	—	1.00	(Ref.)	—			
2	0.95	(0.74–1.21)	0.683	1.00	(0.90–1.12)	0.867			
3 or above	1.16	(0.97–1.39)	0.108	1.14	(1.05–1.23)	0.001			
Histopathology									
Adenocarcinoma	1.00	(Ref.)	—	1.00	(Ref.)	—	1.00	(Ref.)	—
Squamous	1.21	(1.01–1.45)	0.042	1.11	(1.03–1.21)	0.011	1.24	(1.12–1.38)	0.001
Clinical disease stage									
Stage 0	1.00	(Ref.)	—				1.00	(Ref.)	—
Stage I	1.23	(0.46–3.30)	0.677				0.99	(0.54–1.82)	0.970
Stage II	1.64	(0.66–4.09)	0.291				1.47	(0.84–2.57)	0.183
Stage III	2.36	(0.95–5.84)	0.064				2.00	(1.15–3.48)	0.014
Stage IVa	2.41	(0.95–6.12)	0.064				2.42	(1.37–4.27)	0.002
Stage IVb	3.93	(1.58–9.71)	0.003				3.18	(1.83–5.52)	<0.001
Year of diagnosis									
2005–2009	1.00	(Ref.)	—	1.00	(Ref.)	—	1.00	(Ref.)	—
2010–2013	1.64	(1.31–2.06)	<0.001	1.12	(1.03–1.23)	0.013	1.17	(1.03–1.32)	0.017
2014–2018	1.67	(1.32–2.12)	<0.001	1.07	(0.97–1.19)	0.173	1.15	(1.00–1.31)	0.045
Treatment allocation									
Curative treatment	1.00	(Ref.)	—	1.00	(Ref.)	—	1.00	(Ref.)	—
Palliative treatment	2.28	(1.79–2.90)	<0.001	3.19	(2.87–3.54)	<0.001	2.59	(2.28–2.94)	<0.001

Clinical T-, N- and M-stage calculated according to the 8th edition of the AJCC/UICC TNM classification system

Association with improved outcome

Association with poorer outcome

In the survival analysis exploring immortal time bias with the index date moved 21 days forward there were no significant differences from the main analysis.

## DISCUSSION

This nationwide study on patients diagnosed with esophageal cancer finds a clear association between MDT meetings and improved survival, in particular for older patients and patients with advanced disease stage and comorbidities. In addition, the study finds that low socioeconomic status, older age, adenocarcinoma histology and locally advanced clinical tumor stage reduce the likelihood of the patient being presented at an MDT. The availability of the MDT in Sweden has increased over the study period.

MDT meetings allow the patients to be assessed by physicians with different expertise (surgeons, oncologists, radiologists and pathologists), and also adding information attained by nurses, which may include the patient’s social context and performance status. This joint discussion increases the chance for a more diverse evaluation and reduces the need for referrals between participating experts, which might otherwise delay treatment. The oncological field of esophageal cancer treatment is rapidly changing, and active involvement of oncologists, surgeons, radiologists and pathologists is essential to provide the patient a tailored and updated therapeutic approach. Such active involvement in multidisciplinary meetings is greatly influential and has previously been shown to be able to alter diagnostic work-up and/or treatment plans in up to one-third of esophageal cancer cases.^15^ To our knowledge there are no randomized studies evaluating MDT for esophageal cancer patients and as most centers now use MDTs it is unlikely that such a study will ever be undertaken. Observational, non-randomized studies have shown that after implementation of MDT in esophageal cancer, accuracy of staging has improved,^16^ adverse events of neoadjuvant treatment have decreased^17^ and accrual to clinical trials has improved.^18^ Three studies have presented results on survival after implementation of MDT in esophageal cancer. A British study by Stephens *et al*.^6^ showed a markedly improved 5-year survival after MDT introduction for patients undergoing curative resection albeit the referenced study included only 130 patients, was of retrospective design and described a more historical population and setting. A more recent study from Taiwan on esophageal cancer patients, diagnosed between 2010 and 2015, showed a 27% reduced risk for death among MDT participants; however, this study included only patients who allocated to curatively intended treatment.^7^ The same year, 2021, a study from the Netherlands displayed that the 3-year overall survival increased from 24% to 30% for all patients with esophageal cancer after implementation of MDT, and this study comprised increased survival estimates for both curative and palliative patients.^19^ The present study, indicating a 28% reduced mortality risk among cases presented at an MDT were in keeping with the above referenced study findings.

There is no consensus on which investigations should precede the MDT conference and there is a goal conflict in presenting a patient early after diagnosis yet with enough information available to provide an optimal treatment recommendation. MDT conferences may allow patients to have their cases processed in a more streamlined way. In breast cancer, MDT conferences have in this way shortened the delay to treatment^20^ and have also been found to lead to a reduced risk of breast cancer recurrence.^21^ In Sweden, MDT meetings have, over the last two decades, become the norm when assessing patients newly diagnosed with esophageal cancer, and the coverage of the MDTs is monitored in the national esophageal cancer audit. It has taken time for the format to become established as standard of care, but the proportion of patients discussed at an MDT is a recognized marker of quality when Swedish hospital’s care are being audited. This explains why the proportion of patients discussed in MDT has increased over time and in 2018 more than 95% of all Swedish esophageal cancer patients were discussed at an MDT conference.

When analyzing subsets of patients belonging to hypothesized risk groups, we could further explore the associations. Older patients had improved survival when presented to an MDT. This finding was equally identified in patients with more severe comorbidities, in patients with stage IV disease and even in patients with all of the above hypothesized risk factors. This could indicate that MDTs are particularly important for these high-risk patient groups but could also be the effect of selection bias. However, discussing the patient from a multidisciplinary point of view may increase the likelihood that the patient receives a more active palliative treatment plan rather than best supportive care, as supported by Luijten *et al*.,^19^ which was corroborated by the present study findings that active palliation was more often planned in palliative patients who were presented at an MDT. Improved staging may be another explanatory mechanism for improved survival in older and comorbid patients or patients with advanced disease. This is illustrated by the fact that missing information on T-, N- or M-stage was much less common in patients discussed at an MDT. It is also conceivable that bringing the patient forward in an MDT conference raises the expectations on the health care services and possibly the levels of ambition on all levels of health care, which might contribute to prolonged survival for older, comorbid patients or patients with end-stage disease. This is a concerning finding as older patients, or patients with advanced disease, who may not tolerate demanding curative treatment but instead might benefit from palliative treatment, seem to be at a disadvantage in being presented less often to an MDT. Limited availability of the MDT may play a part in this aspect due to misdirected efforts to reduce the workload of any MDT. Clearly palliative patients or older patients deemed unfit for treatment, by the diagnosing physician, might not have been referred to an MDT and were instead offered less tailored palliative treatment, which could explain some of the findings of the study. Notably, patients with advanced disease or many comorbidities had poorer outcome if diagnosed in the latter years of the present study, which might be due to more patients, even patients with poor prognosis were discussed in later MDTs.

Socioeconomic factors such as educational level and being married have previously been shown to increase the chance of receiving curative treatment,^22^ perhaps this is initiated by being presented at an MDT conference. Patients with high educational level may be more vocal concerning their needs and having a spouse or likewise attending visits with and supporting the patient might increase ambition from the health care provider as well as the possibility for the patient to complete an active and demanding tumor treatment.

Strengths of the study are the nationwide coverage and validity^8^ of the registry for patients with esophageal cancer including details on patient, tumor and treatment as well as the prospective collection of data to the NREV and near complete follow-up of patients owing to cross linking with national population registries. The major limitation of the study is the inability to fully control for residual confounding and additional factors that influences both the likelihood of a patient being presented at an MDT and the patient’s survival, and thus might act as a confounding factor. Such a confounder could, for example, be that of two patients with locally advanced cancer, one might have a less advanced disease and better performance status although the TNM stage and Eastern Cooperative Oncology Group (ECOG) performance score may have been reported identical in the registry. It is more likely that the patient with less advanced disease or the more fit patient will be presented at an MDT conference, which could impact our results and must be heeded when drawing conclusions from the study. The centralization of esophageal cancer and the benefits thereof may be closely linked to the infrastructure for and the availability of well-kept MDTs, leading to synergistic effects in cancer care and outcomes. Additionally, improvements in healthcare over time, other than the advancements and benefits of the MDT, may also have influenced the study findings and any potential residual confounding, in this aspect, could not be completely ruled out even though adjustment for year of diagnosis was included in the multivariable models. A risk of immortal time bias was also introduced since all patients presented at an MDT must have survived from diagnosis until the MDT. However, the delay from diagnosis until MDT is fairly short and no significant changes were seen after changing the start of the observation to a time point when a presentation at an MDT had most likely been performed.

The proportion of missing variables, especially regarding staging, was also higher in the no MDT group, which can be viewed as both a weakness as well as a possible explanatory mechanism for some of the study findings, as mentioned above.

## CONCLUSION

The present nationwide study demonstrates a survival benefit for esophageal cancer patients who are discussed in a multidisciplinary setting as part of their treatment pathway. Patients discussed at an MDT lived in median more than 6 months longer than patients who were not presented at an MDT, a more than a two-fold increase in survival. The reasons for this are thought to be multifactorial, and patient selection is significant. Furthermore, the study concludes that older patients, patients with advanced tumor stage and patients with poor socioeconomic status are at a disadvantage because they are less likely to be discussed at an MDT. This finding is especially troublesome because the present study also shows survival benefits for patients in the above risk groups who were discussed in a multidisciplinary setting.

## Supplementary Material

Supplemantary_Table_1_doae061

## Data Availability

All data supporting the results in the paper can be made available upon request.

## Notes

Financial support: Gustav Linder received a general research grant from the Ihre Foundation. Funding bodies had no role in study design, data collection, data analysis, manuscript preparation and publication decisions. All authors had complete access to the study data that support the publication.

Potential competing interests: None.
